# Oxidative Stress and Inflammation, MicroRNA, and Hemoglobin Variations after Administration of Oxygen at Different Pressures and Concentrations: A Randomized Trial

**DOI:** 10.3390/ijerph18189755

**Published:** 2021-09-16

**Authors:** Gerardo Bosco, Matteo Paganini, Tommaso Antonio Giacon, Alberto Oppio, Alessandra Vezzoli, Cinzia Dellanoce, Tatiana Moro, Antonio Paoli, Federica Zanotti, Barbara Zavan, Costantino Balestra, Simona Mrakic-Sposta

**Affiliations:** 1 Department of Biomedical Sciences, University of Padova, 35131 Padova, Italy; tommasoantonio.giacon@studenti.unipd.it (T.A.G.); alberto.oppio@studenti.unipd.it (A.O.); tatiana.moro@unipd.it (T.M.); antonio.paoli@unipd.it (A.P.); 2 Institute of Clinical Physiology, National Research Council (CNR), 20162 Milan, Italy; alessandra.vezzoli@cnr.it (A.V.); dellanoce@ifc.cnr.it (C.D.); simona.mrakicsposta@cnr.it (S.M.-S.); 3 Department of Medical Sciences, University of Ferrara, 44121 Ferrara, Italy; federica.zanotti@unife.it (F.Z.); zvnbbr@unife.it (B.Z.); 4 Environmental, Occupational, Ageing (Integrative) Physiology Laboratory, Haute Ecole Bruxelles-Brabant (HE2B), 1180 Brussels, Belgium; costantinobalestra@gmail.com

**Keywords:** oxygen, redox state, hyperbaric oxygen therapy, hyperoxia

## Abstract

Exercise generates reactive oxygen species (ROS), creating a redox imbalance towards oxidation when inadequately intense. Normobaric and hyperbaric oxygen (HBO) breathed while not exercising induces antioxidant enzymes expression, but literature is still poor. Twenty-two athletes were assigned to five groups: controls; 30%, or 50% O_2_; 100% O_2_ (HBO) at 1.5 or 2.5 atmosphere absolute (ATA). Twenty treatments were administered on non-training days. Biological samples were collected at T0 (baseline), T1 (end of treatments), and T2 (1 month after) to assess ROS, antioxidant capacity (TAC), lipid peroxidation, redox (amino-thiols) and inflammatory (IL-6, 10, TNF-α) status, renal function (i.e., neopterin), miRNA, and hemoglobin. At T1, O_2_ mixtures and HBO induced an increase of ROS, lipid peroxidation and decreased TAC, counterbalanced at T2. Furthermore, 50% O_2_ and HBO treatments determined a reduced state in T2. Neopterin concentration increased at T1 breathing 50% O_2_ and HBO at 2.5 ATA. The results suggest that 50% O_2_ treatment determined a reduced state in T2; HBO at 1.5 and 2.5 ATA similarly induced protective mechanisms against ROS, despite the latter could expose the body to higher ROS levels and neopterin concentrations. HBO resulted in increased Hb levels and contributed to immunomodulation by regulating interleukin and miRNA expression.

## 1. Introduction

Oxygen is the fundamental component of cellular aerobic metabolism. By evolving at 1 atmosphere absolute (ATA), human cells have developed the most efficient way to use oxygen and, at the same time, to protect themselves from its byproducts. Among these, reactive oxygen species (ROS) represent an important family of molecules produced during mitochondrial respiration acting as signaling molecules with regulatory roles on cell activities [1].

At basal rate, it is estimated that 0.2% to 2% of oxygen consumed by mitochondria results in ROS production [2]. Skeletal muscle contraction is related to ROS production [3] as well as the onset of skeletal muscle fatigue [4] and age-related pathological conditions in skeletal muscle. In fact, during exercise, ROS levels increase and create an imbalance in redox status towards oxidation, potentially leading to intracellular damage [5]. ROS generation depends on exercise duration and intensity, gender, and nutritional status, but can be influenced also by individual fitness condition and training level as already demonstrated on animals [6].

While regular training induces beneficial effects by stimulating the expression of antioxidant mechanisms, an inadequately intense exercise such as strenuous marches can be detrimental for health [7]. Acute, intense, and prolonged exercise has been shown to increase oxidative stress [8,9] as well as pro- and anti-inflammatory cytokines, cytokine inhibitors, and chemokines (TNF-α, IL-1β, IL-1ra) [10,11]. Moreover, nitric oxide (NO) production is increased during exercise to allow better vascularization and function of skeletal muscle by modulating microcirculation and hormones [12]. Consequently, the superoxide anion (O_2_^−^) can react with nitric oxide (NO) producing aggressive reactive nitrogen specie (RNS) triggering further ROS formation and reducing NO bioavailability. On the other hand, moderate exercise has proved to reduce oxidative stress onset, activating the endogenous antioxidant defenses [13] and reducing macromolecule damage [14].

ROS and RNS can also increase when physical activity is performed in hyperbaric conditions, such as rebreather diving or breath-hold diving [15,16]. Conversely, training and oxygen pre-breathing at pressure is demonstrated to ameliorate oxidative stress (OxS) [17,18], probably inducing the expression of catalase [19,20], glutathione peroxidase [19,20,21], and superoxide dismutase [20,22].

Recent studies have shown that several microRNA (miRNA) are involved in crosstalk with oxidative stress through ROS regulation [23,24]. Training of both animals and humans in hyperbaric conditions was shown to induce peculiar mRNA expression in muscles, to modulate maximal exercise capacity and physical performance [25,26,27,28,29], and in some cases to help and fasten recovery from damage and fatigue [30]. Additionally, hyperbaric oxygen (HBO) treatments administered after exercise reduced inflammation and oxidative stress [26].

A long tradition of studies highlighted the different mechanism through which oxygen partial pressures (FiO_2_) higher than ambient air can improve exercise performance, but adaptations to chronic administration of higher FiO_2_ are still inconclusive [31]. Additionally, the effects of chronic and intermitted oxygen administration have been studied in the past, especially regarding the mechanisms of hemoglobin increase through the normobaric oxygen paradox [32]. However, reliable data on the best FiO_2_ and long-term consequences are still lacking.

Given these premises, the aim of this preliminary study was to investigate the potential effects of oxygen administered at different concentrations and pressures on oxidative stress, inflammation, microRNA (miRNA) expression, and hemoglobin.

## 2. Materials and Methods

This study was a randomized, patient-blinded, controlled trial (clinicaltrials.gov reg. No. NCT04366427) performed at the physiology laboratories of the University of Padova (Padova, Italy) and at the Domus Medica hyperbaric facility in San Marino (Cittá di San Marino, San Marino Republic).

### 2.1. Selections of Participants

Subjects were recruited through public announcements, without gender restrictions, and considered eligible if complying with the following criteria:

- aged between 18 and 50 years;
- active recreationally athlete: subject involved in a programmed training routine in different mixed sports requiring 3/4 training sessions/week at a medium intensity of 70% of maximal heart rate (calculated with 211 − 0.64 × age [33]) measured by a commercial heart rate monitor (Polar M430, Polar Electro Inc., Kempele, Finland);
- non-smoker.

Before the inclusion, the subjects enrolled passed a general medical exam. Previous pneumothorax or seizures, issues with middle-ear compensation maneuvers, and pregnancy at the moment of inclusion or in the previous 12 months were considered exclusion criteria.

### 2.2. Experimental Protocol

After inclusion, subjects were randomized in five Arms using an electronic number generator by personnel not directly involved in the experiment:

- Arm 1 (control): no intervention;
- Arm 2 (30% O_2_): breathing normobaric air mixture with 30% oxygen for 40 min (at rest);
- Arm 3 (50% O_2_): breathing normobaric air mixture with 50% oxygen for 40 min (at rest);
- Arm 4: treated with 100% oxygen at 1.45 atmosphere absolute (ATA) (hereafter: 1.5 ATA) for 60 min (2 periods of 25 min each, separated by air breaks of 5 min; inclusive of compression and decompression times);
- Arm 5: treated with 100% oxygen at 2.45 ATA (hereafter: 2.5 ATA) for 90 min (3 periods of 25 min each, separated by air breaks of 5 min each; inclusive of compression and decompression times).

Participants were blinded to oxygen concentrations only when using mixtures (Arms 2 and 3).

Subjects included in Arm 1 did not undergo any intervention. Participants in all the other arms underwent a total of 20 treatments (maximum 4 treatments per week, not in the weekends), alternating days of training with days of treatment. All the participants followed a personalized diet proportional to their energetic expenditure.

### 2.3. Measurements and Data Collection

Standard anthropometric parameters were registered at the medical screening (T0) and at the end of the follow up (T2). Blood samples were collected before (T0), at the end (T1), and one month after the end of the treatments (T2); while, urine and saliva samples were collected before (T0), every 7 days during the treatment, at the end of the treatments (T1), at 15 days after T1, and at one month after the end of treatments (T2). A graphical representation of the protocol timeline is available as Supplementary Materials, Figure S1.

Approximately 13 mL of venous human blood was drawn from an antecubital vein, with subjects sitting or lying on a bed. Samples were collected in one lithium-heparinized and one EDTA tube (Vacutainer, Becton Dickinson, Franklin Lakes, NJ, USA). Plasma was separated by centrifuge (5702R, Eppendorf, Germany) at 3500 rpm for 10 min at 4 °C. All samples were then stored in multiple aliquots at −80 °C until assayed. Samples were thawed only once before analysis, performed within one month from collection.

Approximately 1 mL of saliva was obtained by Salivette devices (Sarstedt, Nümbrecht, Germany). The subjects were instructed on the correct use of the devices [34]. Samples were spun down, aliquoted, and stored.

Urine was collected by voluntary voiding in a sterile container (20 mL) and stored in multiple aliquots at −20 °C until assayed and thawed only before analysis.

#### 2.3.1. Oxidative Stress and Oxidative Damage

An X-band Electron Paramagnetic Resonance instrument (E-Scan-Bruker BioSpin, GmbH, Billerica, MA USA) was adopted. ROS production rate and antioxidant capacity (TAC) were determined as already performed on blood and saliva [16,35,36]. CMH (1-hydroxy-3-methoxycarbonyl-2,2,5,5-tetramethylpyrrolidine) and DPPH (2,2-diphenyl-1-picrylhydrazyl—a free radical compound soluble and stable in ethanol) spin probe and traps were used, respectively, for determined ROS and TAC. A stable radical CP· (3-Carboxy-2,2,5,5-tetramethyl-1-pyrrolidinyloxy) was used as external reference to convert ROS determinations in absolute quantitative values (μmol/min), while TAC was expressed in terms of Trolox equivalent (mM). A controller “Bio III” unit, interfaced to the spectrometer, was used to stabilize sample temperature at 37 °C. Samples were analyzed in duplicate. All EPR spectra were collected by adopting the same protocol and obtained by using a software standardly supplied by Bruker (Billerica, MA USA) (version 2.11, Win EPR System).

#### 2.3.2. Isoprostane

Lipid peroxidation was measured on urine by immunoassay of 8-isoprostane concentration (8-iso-PGF2 α) (Cayman Chemical, Ann Arbor, MI, USA) as previously described [16,37]. Samples were read in duplicate at a wavelength of 512 nm.

#### 2.3.3. Nitrite and Nitrate Levels (NOx)

NOx concentrations were determined on urine via a colorimetric method based on the Griess reaction, using a commercial kit (Cayman Chemical, Ann Arbor, MI, USA) as previously described [16,38]. Samples were read in duplicate at 545 nm.

#### 2.3.4. Inducible Nitric Oxide Synthase (iNOS)

To assess inducible nitric oxide synthase (iNOS) expression in plasma, a human NO_2_/iNOS ELISA kit (cat no EH0556; FineTest, Wuhan, China) was used. This assay was based on sandwich enzyme-linked immune-sorbent assay technology. NOS_2_/iNOS protein synthesis was determined using a standard curve. Samples and standards were read at a wavelength of 450 nm, and the analysis was carried out according to the manufacturer’s instructions.

#### 2.3.5. Inflammatory Status

Interleukins IL-6 (Cayman Chemical, Ann Arbor, MI, USA, Item No. 501030), IL-10 (Cayman Chemical, Ann Arbor, MI, USA, Item No. 589201), and TNF-α (ThermoFisher Scientific, Waltham, MA, USA) Item No. EHIL10) plasmatic levels were measured using human interleukins ELISA kits, according to the manufacturer’s instructions. The determinations were assessed in duplicate, and the inter-assay coefficient of variation was in the range indicated by the manufacturer.

All the colorimetric and immune enzymatic assays were read by a microplate reader spectrophotometer (Infinite M200, Tecan Group Ltd., Männedorf, Switzerland).

#### 2.3.6. Thiols

Total (tot) and reduced (red) aminothiols (Cys: cysteine; CysGly: cysteinylglycine; Hcy: homocysteine; and GSH: glutathione) concentrations were measured in red blood cells according to previously validated methods [39,40]. Briefly, thiols separation was performed at room temperature by isocratic HPLC analysis on a Discovery C-18 column (250 × 4.6 mm I.D, Supelco, Sigma-Aldrich, St. Louis, MO, USA), eluted with a solution of 0.1 M acetate buffer, pH 4.0: methanol, 81:19 (*v*/*v*), at a flow rate of 1 mL/min. Fluorescence intensities were measured with an excitation wavelength at 390 nm and an emission wavelength at 510 nm, using a fluorescence spectrophotometer (Jasco, Tokyo, Japan). A standard calibration curve was used.

#### 2.3.7. Creatinine, Neopterin and Uric Acid Concentration

Creatinine and neopterin urinary concentrations were measured by high-performance liquid chromatography (HPLC) method as previously described [16]. Additionally, uric acid levels were determined by Varian instrument (pump 240, autosampler ProStar 410, Varian Medical Systems Inc., Palo Alto, CA, USA) coupled to a UV-VIS detector (Shimadzu SPD 10-AV (Shimadzu Corporation, Kyoto, Japan), λ = 240 nm) after centrifugation at 13,000 rpm for 5 min at 4 °C. Analytic separations were performed at 50 °C on a 5 µm Discovery C18 analytical column (250 × 4.6 mm I.D., Supelco, Sigma-Aldrich, St. Louis, MO, USA) at a flow rate of 0.9 mL/min. The calibration curves were linear over the range of 0.125–1 μmol/L, of 3.75–60 mmol/L, and of 1.25–10 mmol/L for neopterin, uric acid and creatinine levels, respectively. Inter-assay and intra-assay coefficients of variation were <5%.

#### 2.3.8. Total RNA and miRNA Isolation

Total miRNAs were isolated with miRNeasy Mini Kit (Qiagen GmbH, Hilden, Germany) from plasma samples. NanoDropTM ND-1000 (Thermo Fisher Scientific, Waltham, MA, USA) was used to assess quality and concentration of the RNA samples. Complementary DNA (cDNA) was obtained from 200 ng of total RNA using M-MLV Reverse Transcriptase (Invitrogen, Carlsbad, CA, USA) or miRcute miRNA First-strand cDNA Synthesis Kit (Tiangen Biotech, Shangai, China), following the manufacturer’s protocols. Real-time PCRs were performed with a Rotor-Gene 3000 (Corbett Research, Sydney, Australia) using 300 nM concentration of primers and FastStart SYBR Green Master (Roche Diagnostics, Mannheim, Germany). Differences in gene expression were evaluated by the 2∆∆Ct method, [41] using plasma derived from control sample. The mature miRNAs expression levels were estimated with the miRcute miRNA qPCR detection kit (Tiangen Biotech, Shangai, China). The relative miRNA levels were calculated by the 2∆∆Ct method after normalization to snRNA-U6 expression.

#### 2.3.9. Hemoglobin

Hemoglobin concentration was determined from lysed RBCs by irreversible reaction with potassium cyanide and potassium ferricyanide, and oxidized to the stable pigment cyanmethemoglobin by Drabkin’s method. Thawed erythrocytes are diluted 1:2 (*v*/*v*) with distilled water and then refrozen. The samples are thawed again and 10 µL of sample is placed in each well of the plate to which 190 µL of Drabkin’s solution is added. After 30 min, the absorbance is read spectrophotometrically at 540 nm and is directly proportional to the hemoglobin concentration. An Hb standard calibration curve was used starting with a stock solution of 40 mg/mL [42].

### 2.4. Modifications to the Protocol

After protocol registration but before enrollment of subjects, miRNA dosage was added to the investigations performed. Additionally, saliva and urine measures were performed more frequently during HBO treatments as being noninvasive and to depict the variations in a more precise manner. VO_2_MAX testing with lactate clearance was not performed due to COVID-19 restrictions on experiments.

### 2.5. Statistical Analysis

Statistical analyses were performed using the software Prism 9 (GraphPad Prism 9.2.0, Software Inc., San Diego, CA, USA). Taking the baseline measures as reference (100%), percentage variations ((post-pre/pre value) × 100) were calculated for each condition, allowing an appreciation of the magnitude of change rather than the absolute values. After the Shapiro–Wilk and D’Agostino-Pearson normality test, statistical analyses were performed. One sample *t*-test, with hypothetical value at 100% were performed.

The Mann–Whitney test was adopted to compare same biomarker at same time in different group (i.e., ROS at T1 between 1.5 vs. 2.5 ATA HBO).

Time course of Reactive Oxygen Species, Antioxidant capacity and lipid peroxidation were analyzed with a one-way ANOVA for repeated measures with Dunnett’s post hoc test. Results are expressed as percentage ± SD and significant difference was set at *p* < 0.05.

## 3. Results

A total of 22 subjects (3 females, 19 males) were included in this study: controls, *N* = 6; 30% O_2_, *N* = 3; 50% O_2_, *N* = 4; 1.5 ATA, *N* = 6; 2.5 ATA, *N* = 3.

Anthropometric characteristics (age, weight, height), and clinical hematological parameters are reported in Table 1 to confirm the absence of pathological condition at the moment of the inclusion, without variation along the study.

Outcome values of subjects included in Arm 1 (controls) remained stable during the considered period of time, therefore only T0 has been reported.

### 3.1. Oxidative Stress, Nitric Oxide, and Inflammation Status

**Figure 1:**
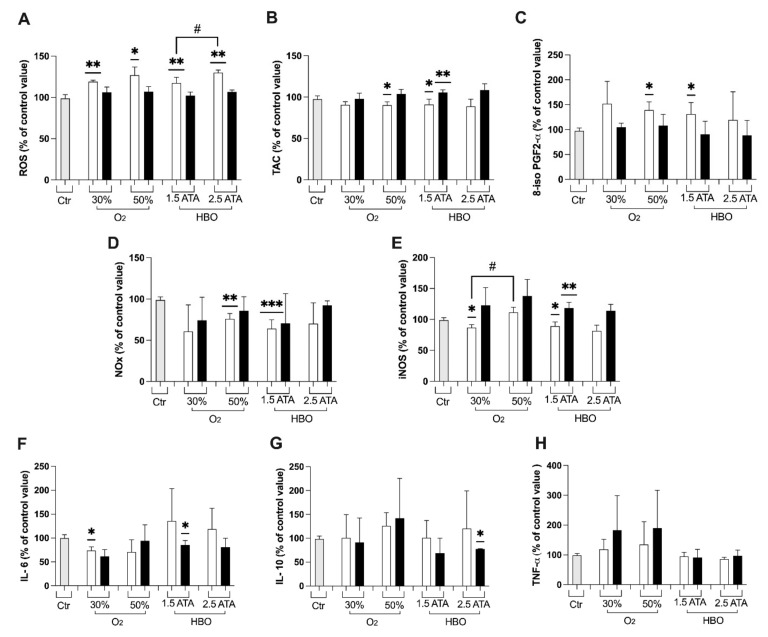
Histogram plot (% ± SD) of (**A**) Reactive Oxygen Species (ROS), (**B**) Total Antioxidant Capacity (TAC), (**C**) 8-isoprostane (8-iso PGF-2α), (**D**) NO metabolites (NOx), (**E**) iNOS, (**F**) IL-6, (**G**) IL-10, and (**H**) TNF-α, obtained from plasma samples collected. The control group (Ctr) is reported in grey; white and black bars identify T1 and T2, respectively. Significant differences intra-group: * *p* < 0.05; ** *p* < 0.01; *** *p* < 0.001. Significant difference between groups: # *p* < 0.05.

In both the groups breathing O_2_ mixtures at 30% and 50% at rest (Arms 2 and 3), higher ROS levels were registered at T1 (Figure 1A), along with a decrease in TAC (Figure 1B), an increase in lipid peroxidation (Figure 1C), and a decrease in NO metabolites (NOx, Figure 1D). Additionally, both these treatments induced up-down regulation of inducible nitric oxide synthase (iNOS) enzyme transcription in T1and T2 (Figure 1E). Concordantly with these results, a slight rise of inflammation through cytokine levels (Il-10, TNF-α), (Figure 1G,H) was detected despite not statistically significant. Only IL-6 (Figure 1F), significantly decreased at T1.

At T1, HBO treatment at 1.5 ATA (Arm 4) showed significant increases in oxidative stress biomarkers (ROS, TAC and 8-isoPGF2α; Figure 1A–C), NOx (Figure 1D), and iNOS (Figure 1E), but not significant changes in the inflammatory status (Il-6, Il-1β and TNF-α; Figure 1F–H). By contrast, a significant drop in IL-6 and IL-10 at T2 was detected after HBO 1.5 and 2.5 ATA treatments, respectively, when compared to controls.

**Figure 2:**
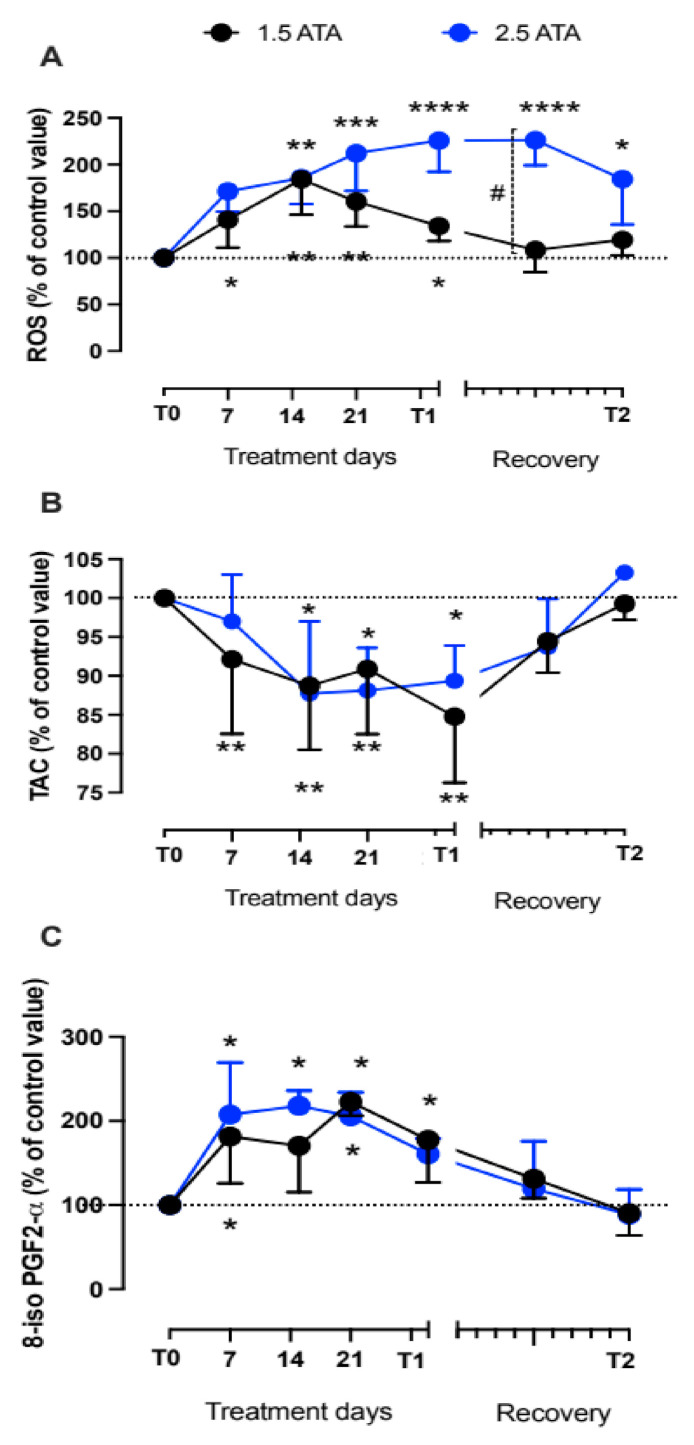
Collected samples during treatments session at 1.5 and 2.5 ATA, and recovery. Time course (% ± SD) of (**A**) Reactive Oxygen Species (ROS) production and (**B**) Total Antioxidant Capacity (TAC) detected on saliva by EPR technique, and (**C**) 8-isoprostane (8-iso PGF2α) measured on urine by immune-enzymatic assay. Significant difference intra-group: * *p* < 0.05; ** *p* < 0.01; *** *p* < 0.001, **** *p* < 0.0001. Significant difference between groups: # *p* < 0.05.

During HBO treatments (1.5 and 2.5 ATA) three parameters of oxidative stress were also monitored on saliva (ROS, TAC) and in urine (8-iso PGF2-α) samples. Both HBO treatments induced detectable changes in ROS, TAC, and 8-iso PGF2-α levels, measured at the different times. The delta (%) concentrations of ROS, TAC, and 8-iso PGF2α calculated at any time and the statistically significant differences between times of measurements are shown in Figure 2A–C. After treatment days, oxidative stress biomarkers levels showed that the redox-balance returned to the baseline levels (100%).

**Figure 3:**
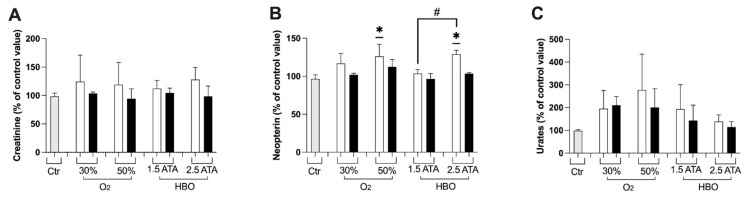
Histogram plot (% ± SD) of renal function biomarkers: (**A**) Creatinine, (**B**) Neopterin and (**C**) Urates. The control group (Ctr) is reported in grey; white and black bars identify T1 and T2, respectively. Significant difference intra-group: * *p* < 0.05. Significant difference between groups: # *p* < 0.05.

Changes (%) in renal function biomarkers—creatinine, neopterin, and urates—are depicted in Figure 3A–C. A similar increase in neopterin was recorded at T1 by breathing 50% O_2_ and HBO 2.5 ATA, followed by a slow return to basal levels. Furthermore, significant differences were observed between HBO treatment 1.5 and 2.5 ATA at T1. No differences were observed in creatinine and urea marker in all treatments.

**Figure 4:**
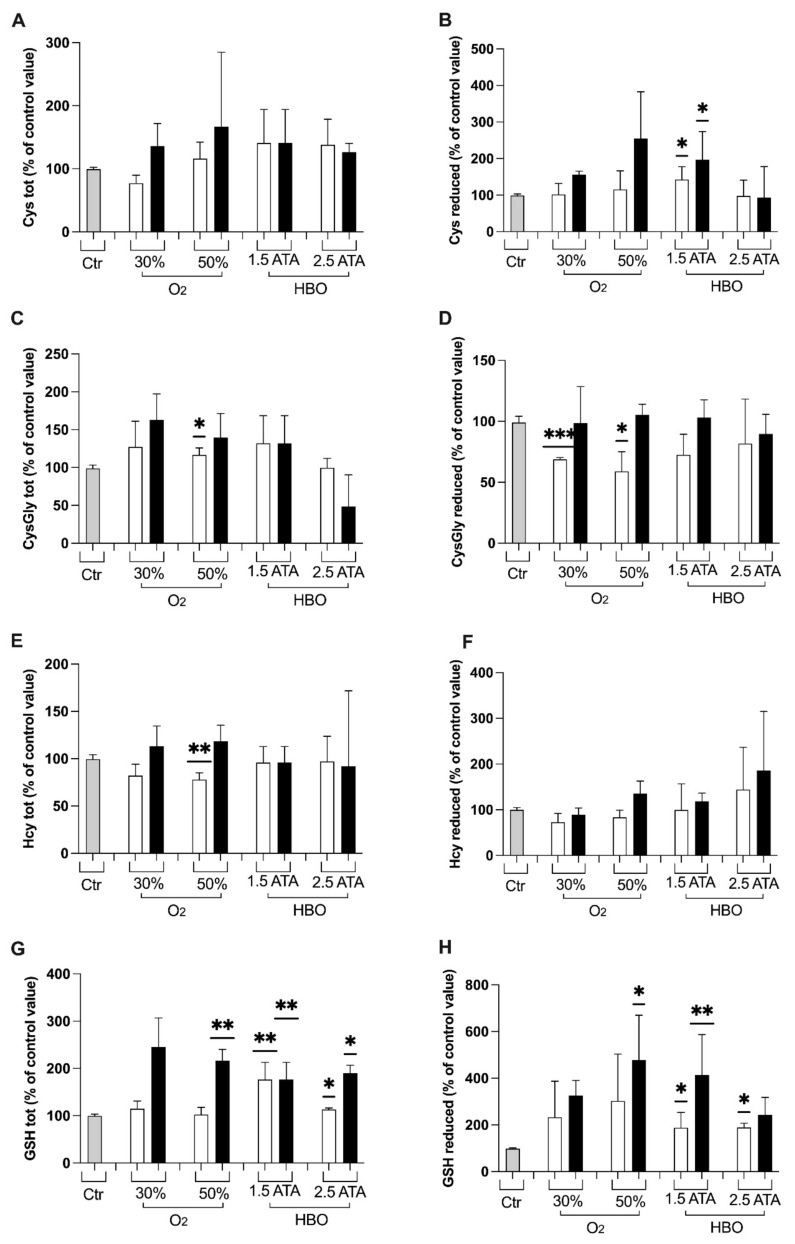
Histogram plot (% ± SD) of total and reduced aminothiols. The control group (Ctr) is reported in grey; white and black bars identify T1 and T2, respectively. Significant difference intra-group: * *p* < 0.05; ** *p* < 0.01; *** *p* < 0.001.

At the end of the treatments with 50% O_2_ mixture, significant changes in total CysGly, Hct and GSH were observed (Figure 4C,E,G). Reduced CysGly concentration was significantly decreased at T1 after both 30% and 50% treatments (Figure 4D).

No significant differences in total aminothiols were detected at T1 and T2 for both HBO treatments, except for GSH at 1.5 ATA (Figure 4G). Higher levels of total and reduced GSH were observed, resulting from a positive shift in redox balance towards a more reduced state (Figure 4G,H). Besides, HBO treatment 1.5 ATA showed a significant increase in reduced Cys and decrease in reduced CysGly. No significant differences between groups were observed.

### 3.2. Circulating miRNA

**Figure 5:**
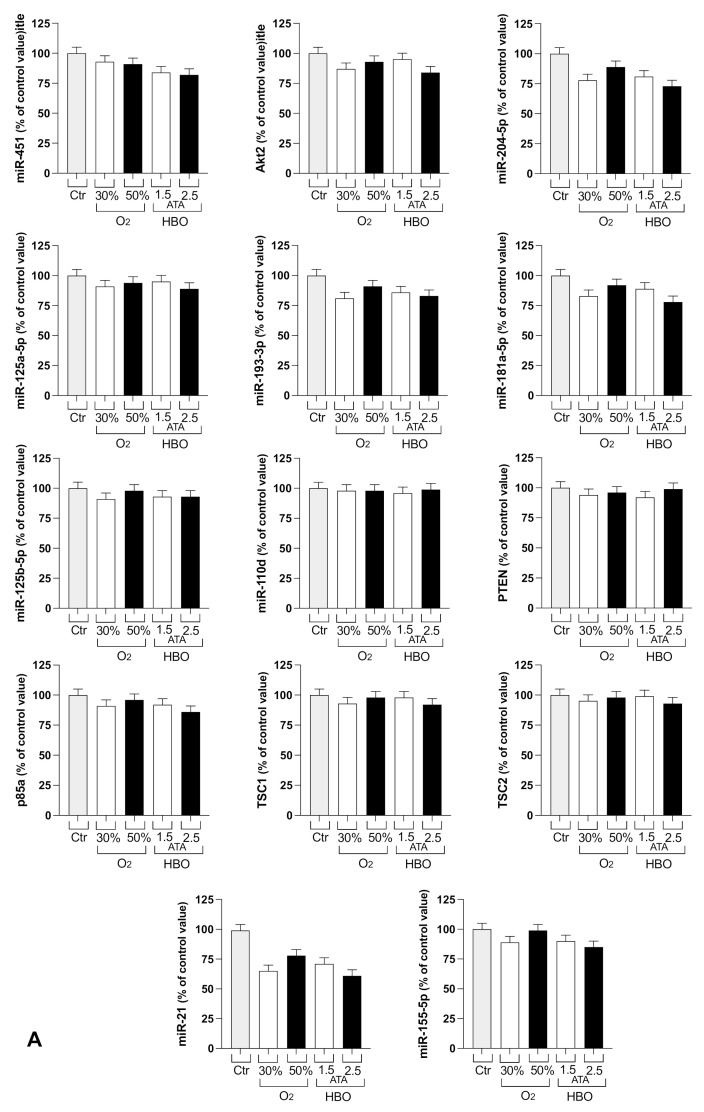

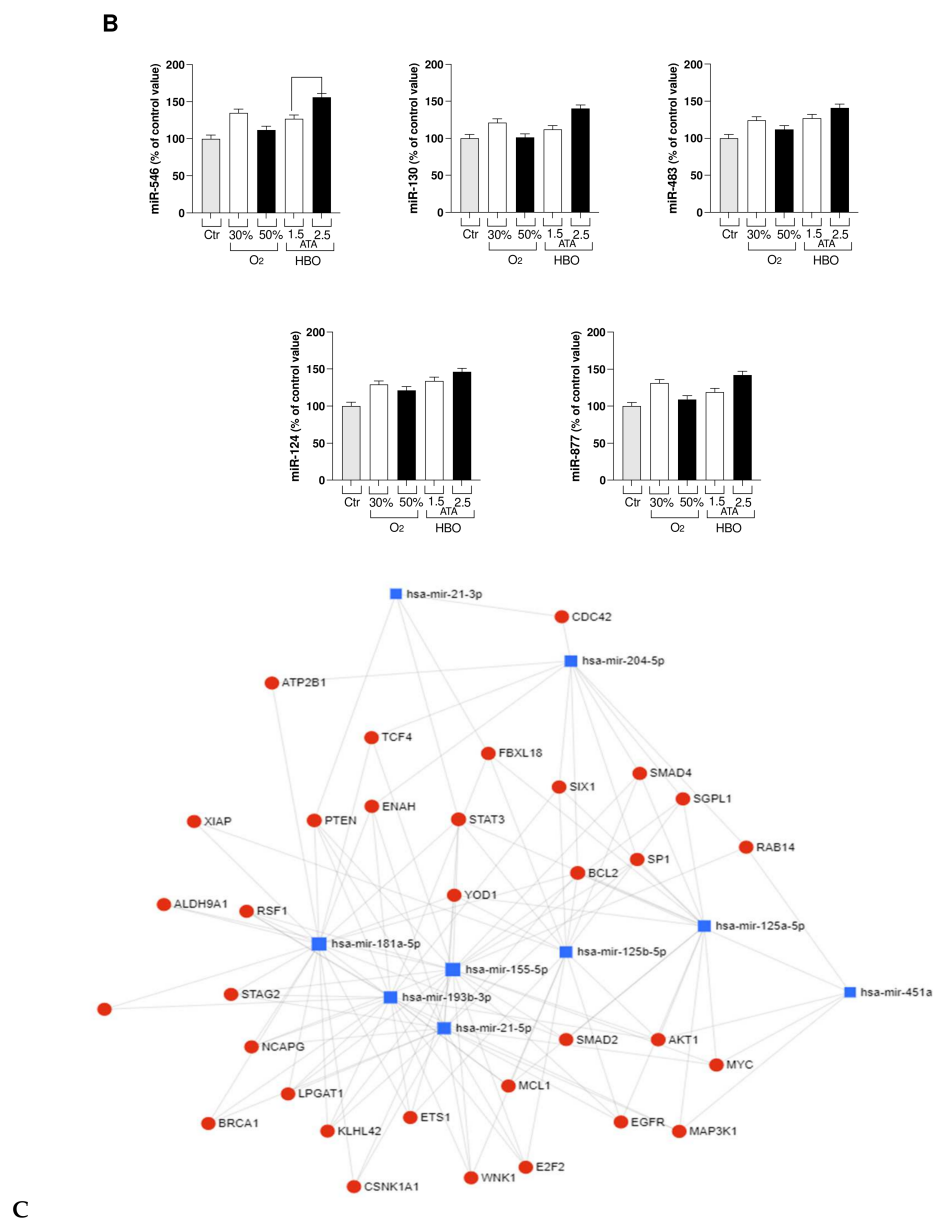
(**A**,**B**) histogram of gene expression related to miRNA involved on macrophages polarization M2 (**A**) and M1 (**B**). Results are reported as % of variation of T2 compared to T1. Control is basal line. Map (**C**) of pathway involved by the miRNA.

Circulating miRNA related to the immunomodulatory effect have been detected by means of PCR real time. Results reported on Figure 5 show a net effect of immunomodulation in the Arms 2 and 3.

Specifically, miRNA related to the macrophage M2 macrophages type associate, to anti-inflammatory phenotype was higher after treatments while miRNA associate to M1-inflammatory phenotype was reduced, in relationship with the increased treatment time and % O_2_ in the mixtures. This means that in presence of increasing of % O_2_ more circulating monocytes should receive more input to be committed into M2 (anti-inflammatory) phenotype.

Among these groups of miRNA, the miRNA124 was the most represented, as being involved with inflammatory processes, as well as with angiogenesis and smooth muscles physiology. As a net result, inflammation was modulated and vascularization improved.

### 3.3. Hemoglobin

**Figure 6:**
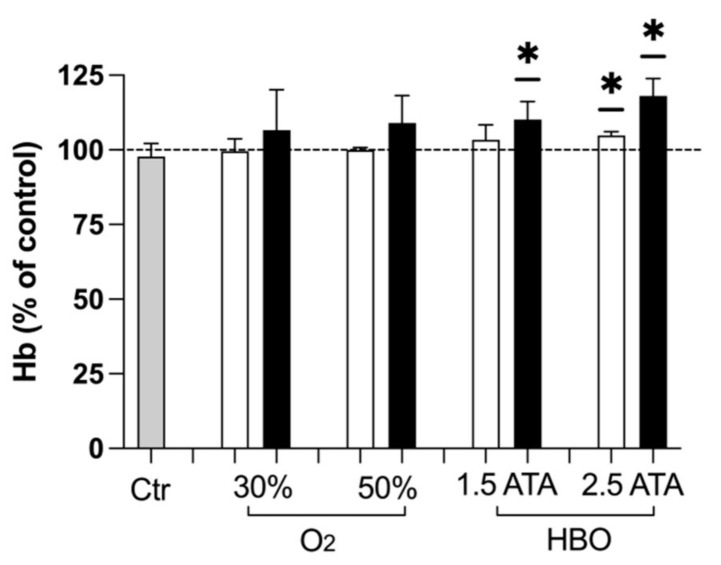
Histogram plot (% ± SD) of red blood cells hemoglobin (Hb). The control group (Ctr) is reported in grey; white and black bars identify T1 and T2, respectively. Significant difference intra-group: * *p* < 0.05. Significantly higher Hb concentration was detected at T2 with both HBO treatments.

In subjects undergoing treatments with O_2_ mixtures, a slight trend towards higher hemoglobin levels was noted, despite being not significant. Instead, such increase reached statistical significance with both HBO treatments at T2 (Figure 6).

## 4. Discussion

With this work, we present the results of a preliminary experiment involving the administration of oxygen at different concentrations and pressures to primarily investigate effects on the oxidative stress panel, and secondarily the effects on inflammation, miRNA expression and hemoglobin in athletes.

ROS are important mediators in several cellular pathways, modulating proliferation, survival, apoptosis, and immune response. One of the major sources of ROS/RNS is the immune system [43], produced by neutrophils [44], as a consequence of the inflammation status related to high-intensity exercise [45]. In fact, ROS produced during exercise promote neutrophils’ muscle infiltration by increasing vascular permeability [46] and could lead to increased muscular damage. ROS also increase when more oxygen is available, as demonstrated from T1 samples in all the intervention arms (Figure 1A).

During hyperbaric hyperoxia, the whole body compensates to accommodate the increased oxygen stimulus and the higher amount of ROS with a number of adaptive mechanisms. A vasoconstriction response helps modulating the amount of oxygen delivered to the brain and tissues [47], while endogenous antioxidant defense systems are enhanced to counterbalance the induced oxidative stress [48]. Consistently, treatments with both 30% and 50% O_2_ mixtures at rest and both HBO at 1.5 and 2.5 ATA demonstrated to increase TAC at T2 (Figure 1B), counterbalancing ROS-related damages.

We found most variation in ROS production. Figure 2 suggests that ROS generation is greater at 2.5 ATA, but the antioxidant capacity and the oxidative damage to lipids appears to be the same, especially 14 days after starting the treatments. Additionally, levels of lipid peroxidation (8-isoprostane) demonstrate a similar kinetic and tend to return into control values (100%) after one month.

Thiols, such as homocysteine (Hcy), cysteine (Cys), and cysteinylglycine (CysGly), are metabolically interrelated antioxidants and can be considered the principal interface with the changing redox environment, able to protect cellular component and involved in cellular homeostasis [39,48,49,50]. Erythrocytes have been used as a simple model to study the cellular effects of ROS and appropriate for intracellular redox status analysis [39]. Overall, the aminothiols balance was preserved (Figure 4). The significant increase in total and reduced GSH after both HBO treatments (T1) observed in erythrocytes suggests that these treatments are similar in inducing a higher imbalance of redox status than hyperoxic mixtures (Figure 4H). Moreover, both HBO at 1.5 and 2.5 significantly increased total GSH levels at follow up, suggesting the protective role of these treatments against oxidative stress (Figure 4G).

Overall, these results seem to suggest that HBO at 1.5 and 2.5 ATA induce a similar response in protective mechanisms against ROS, despite the latter could expose the body to higher ROS levels (Figure 1A) and renal damage predisposition (Figure 3B: higher neopterin levels at T1). Moreover, the increase in total and reduced GSH indicates that redox status has been positively unbalanced towards the reduced state and capable of contrasting ROS damages. Several pathological conditions—such as aging or degenerative diseases—persist in an oxidated environment with decreased levels of GSH. Similarly, the increase in aminothiols has important implications in cardiovascular diseases prevention, but these results should be validated on larger samples.

The involvement of the inflammatory components has an important role in the progression of some metabolic dysfunction/pathologies that may affect the endothelium and other cell types. As well as endothelial activation, they may interfere, for instance, with the production of nitric oxide (NO). We observed a change in inflammatory markers (IL-6-10) at 30% O_2_ and at 1.5 ATA. Our data are in accord with Woo et al. [26], showing that HBO treatment in the recovery phase had a positive impact on relieving the inflammatory response and muscle damage. Furthermore, HBO inhibits stimulus-induced proinflammatory cytokine synthesis by human blood-derived monocyte-macrophages [51]. Based on this, and our results, we can also hypothesize anti-inflammatory effects with 30% O_2_ treatment.

Other experiences in which athletes were exposed to different oxygen concentrations revealed an increase in performance, VO_2_max and Cardiac Output in hyperoxia compared with hypoxia and normoxia [52]. Both mixtures at rest—but mostly the 50% O_2_ mixture—demonstrated to promote the transcription of iNOS at the follow up (Figure 1E)—probably resulting in higher NO levels and, therefore, positive effects on peripheral and pulmonary vascular tone modulation. Additionally, the 50% O_2_ mixture produced a significant increase in total and reduced GSH at the follow up, suggesting again its protective role. Literature shows conflicting results on sea-level performance after chronic training in hyperoxia [53,54,55]. Specifically, transport mechanisms have been proposed as responsible of limiting the increase of aerobic power [53]. However, the findings of the present study suggest the usefulness of hyperoxic mixtures administered to athletes also at rest, especially to those performing exercise at high levels. Further studies should confirm the role in other subsets of subjects.

Growing evidence suggests an important crosstalk between ROS and microRNAs [56]. In particular, recent studies correlated oncogenesis and ROS; specifically, ROS control miRNA expression through epigenetic modifications. ROS inhibit and enhance expression of certain miRNA genes through methyltransferase (DNMT1) and histone deacetylases (HDACs), respectively, and it can also activate transcription factors to induce miRNA expression.

Macrophages are usually the first immune cells to face invading pathogens, and use phagocytosis to degrade microbes, dead cells, or cellular debris in phagolysosomes. Indirect antimicrobial mechanisms include the activation of inflammasomes and the secretion of cytokines and chemokines, which help to orchestrate the subsequent innate and adaptive immune responses. Furthermore, macrophages are fundamental actors of tissue vascularization, regulating both blood and lymphatic vessel growth, specifically after tissue injury or pathological inflammatory responses. Neovascularization depends on immunity and inflammation, but also hypoxia is a strong promoter of angiogenesis. In this process, a key role is represented by miRNAs that are noncoding RNA transcripts and then proteins but regulate cell functions: for example, inducing the polarization of macrophages into M1 or M2. M1 macrophages are the predominant phenotype in normal immunological responses and involved in type I T helper cells response against different pathogens. M1 macrophages also produce pro-inflammatory cytokines with tumor-cell and microbe-killing activities. M2 macrophages instead induce immunosuppression, angiogenesis, elimination of parasites, and are involved in wound repair. Several microRNAs are able to regulate M1 or M2 macrophage-type polarization. In particular, an increase in the parameters related to the anti-inflammatory M2 phenotypes of macrophages and a reduction of the inflammatory M1 phenotype was seen in all the groups, with an increasing trend towards HBO, especially 2.5 ATA.

Moreover, miRNA can be double-faced with expression levels (i.e., miR-21) deleterious actions or, in addition, the protective effects: silencing fall on neovascularization and inflammation in diabetic retinopathy [57]; and recently, has been demonstrated, that circulating microRNA-21 is an early predictor of ROS-mediated damage in patient affect in diabetes type 2 [57]. Future studies should specifically target this field and investigate how to enhance the anti-inflammatory pattern, also in light of a possible role of miRNAs in inhibiting gene expression of SARS-CoV-2 and other viruses [58].

Finally, an increasing trend in hemoglobin levels has been detected after all the treatments at T1 and T2, again significant only in groups undergoing HBO and especially seen in the 2.5 ATA group. Previously, the application of intermittent normobaric oxygen contributed to raise Hb in chronic anemic patients [59,60], and especially when oxygen was administered on alternate days to non-anemic subjects [61]. This study instead suggests that hyperbaric hyperoxia has the greatest effect in increasing Hb levels of non-anemic athletes, probably due to higher oxygen tissue levels reached, but did not clarify the administration time required to achieve such outcome. Further studies should specifically address this topic for its clinical implications in sports medicine, gerontology and respiratory rehabilitation.

This paper has several limitations. First, the small sample of subjects included in the experiments hampers current clinical applications. As these treatments need a solid background in molecular sciences, no clinical or macroscopic outcomes were evaluated at the moment. Moreover, the results could have been affected by unaccounted factors, such as different training schedules and exercises. Therefore, these preliminary findings should be interpreted with caution, but will help in refining future studies in the field.

## 5. Conclusions

The results suggest that HBO at 1.5 and 2.5 ATA similarly induce protective mechanisms against ROS, despite the fact that the latter could expose the body to higher ROS levels and neopterin concentrations. The increase in total and reduced GSH indicates that redox status has been positively unbalanced towards the reduced state and is capable of contrasting ROS damages. Furthermore, HBO resulted in increased Hb levels and contributed to immunomodulation. It may suggest an oxygen induced anti-inflammatory and neoangiogenetic effect due to interleukin and miRNA assessments. In the future, a higher number of subjects involved will shed more light on the studied effects and possible applications.

## Figures and Tables

**Table 1:** Subjects’ anthropometric data and hematological parameters determined at the baseline.

Parameter (Median, IQR)	
Age (years)	37 (33–46)
Weight T0 (kg)	73 (64–79)
Height (cm)	174 (168–180)
Leukocytes (109/L)	6.29 (5.49–8.23)
Erythrocytes (1012/L)	4.98 (4.77–5.39)
Hemoglobin (g/dL)	15.35 (14.00–15.9)
Hematocrit (%)	45.00 (42.78–46.18)
Platelets (109/L)	248.00 (200.50–283.75)
Erythrocyte Sedimentation Rate (mm)	5.50 (3.75–6.50)

## Data Availability

Data are available from the corresponding author upon request.

## Supplementary Materials

The following are available online at https://www.mdpi.com/article/10.3390/ijerph18189755/s1, Figure S1: Timeline of the protocol.
