# Supplementary material for: Oxidative Stress and Inflammation, MicroRNA, and Hemoglobin Variations after Administration of Oxygen at Different Pressures and Concentrations: A Randomized Trial
Source: Int J Environ Res Public Health. 2021 Sep 16;18(18):9755. doi: 10.3390/ijerph18189755 (PMC8468581; doi:10.3390/ijerph18189755)
Supplement: Supplementary file 1 [file ijerph-18-09755-s001.zip › ijerph-1358993-supplementary.pdf]

Oxidative Stress and Inflammation, MicroRNA, and Hemoglobin Variations after Administration of Oxygen at Different Pressures and Concentrations: A Randomized Trial. By Bosco G. et al.

Supplementary Material Figure S1: protocol timeline.

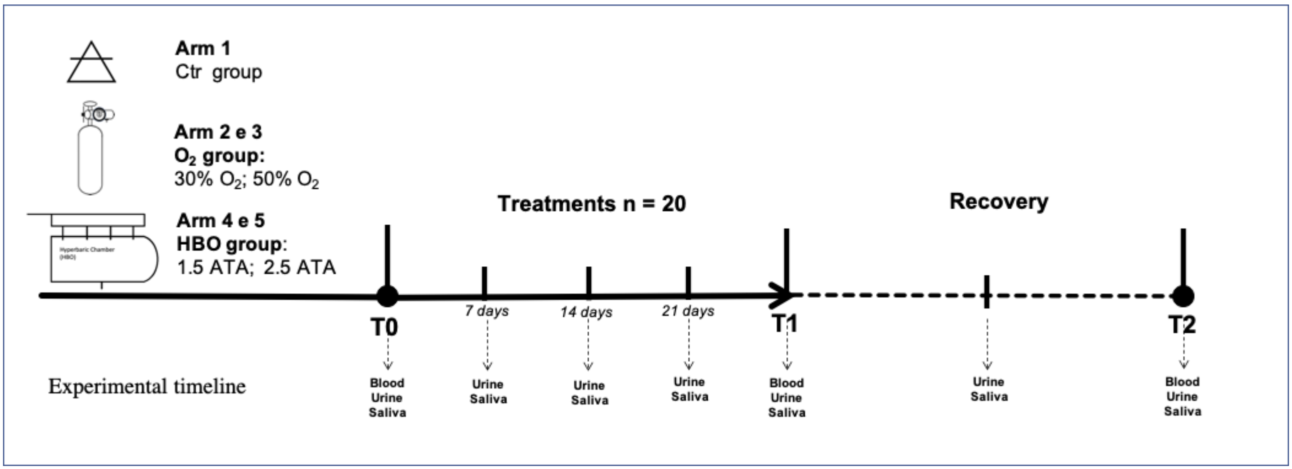

Arm 1: control group
Arm 2: 30% oxygen
Arm 3: 50% oxygen
Arm 4: 1.5 ATA
Arm 5: 2.5 ATA

T0 = before treatments
Treatment time: 20 treatments – 4 times per week
T1 = end of treatments
T2 = one month the end of treatments
